# Research on Rare Diseases in Germany – The GAIN Registry: a registry for individuals with congenital multi-organ autoimmune diseases

**DOI:** 10.25646/11732

**Published:** 2023-12-13

**Authors:** Cynthia Stapornwongkul, Alexandra Nieters, Paulina Staus, Stephan Rusch, Anita Delor, Ulrich Baumann, Julius Wehrle, Melanie Boerries, Markus G. Seidel, Bodo Grimbacher, Gerhard Kindle

**Affiliations:** 1 Institute for Immunodeficiency, Center for Chronic Immunodeficiency (CCI), Medical Center – University of Freiburg, Faculty of Medicine, University of Freiburg, Germany; 2 FREEZE-Biobank, Center for Biobanking, Medical Center – University of Freiburg, Faculty of Medicine, University of Freiburg, Germany; 3 Institute of Medical Biometry and Statistics, Medical Center – University of Freiburg, Faculty of Medicine, University of Freiburg, Germany; 4 Department of Paediatric Pulmonology, Allergy and Neonatology, Hannover Medical School, Germany; 5 Institute of Digitalization in Medicine, Medical Center – University of Freiburg, Faculty of Medicine, University of Freiburg, Germany; 6 Department of Medicine I, Medical Center – University of Freiburg, Faculty of Medicine, University of Freiburg, Germany; 7 Institute of Medical Bioinformatics and Systems Medicine, Medical Center – University of Freiburg, Faculty of Medicine, University of Freiburg, Germany; 8 Division of Pediatric Hematology-Oncology, Department of Pediatrics and Adolescent Medicine, Medical University of Graz, Austria; 9 Department of Rheumatology and Clinical Immunology, Medical Center – University of Freiburg, Faculty of Medicine, University of Freiburg, Germany

**Keywords:** MULTI-ORGAN AUTOIMMUNE DISEASES, RARE DISEASES, QUALITY OF LIFE, CONGENITAL IMMUNODEFICIENCIES

## Abstract

**Background:**

Patient registries are an important tool for networking medical caregivers and research, especially in the field of rare diseases. Individuals afflicted by multi-organ autoimmune diseases typically suffer from inflammation of multiple organs.

**Project:**

GAIN (German genetic multi-organ Auto-Immunity Network) is the German network for research and therapy optimisation for individuals with congenital multi-organ autoimmune diseases. As a sub-project of the network, the registry systematically collects data from this patient group and makes it available for research purposes.

**Results:**

A data set was developed and made available for the GAIN Registry that can map the complex clinical status of persons with multi-organ autoimmune diseases. Data from 486 individuals have been documented to date.

**Conclusions:**

The GAIN register allows for a very comprehensive documentation that clearly goes beyond previous approaches, e.g. by linking it to biosamples collected in the consortium. The planned inclusion of patients in the documentation, e.g. of data on quality of life, opens up a new field.

## Introduction

Congenital multi-organ autoimmune diseases belong to the extremely rare diseases. In multi-organ autoimmune diseases, the body’s immune system mistakenly attacks its own organs. Afflicted people typically experience inflammation in several organs, for example bone marrow, intestines, lungs, kidneys, skin and central nervous system. The complexity of the disease patterns and the insufficient data make it difficult to diagnose and treat the afflicted patients. Monogenic mutations (changes affecting one gene) in genes that regulate the immune system are already known to be a cause. However, the presence of a gene mutation alone does not determine whether the disease actually becomes manifest in a patient. In addition to other as of yet unknown gene mutations, environmental factors such as lifestyle and infections may also play a role in the development of multi-organ autoimmune disease. Research into these diseases may also contribute to the understanding and treatment of more common polygenic (affecting multiple genes) autoimmune diseases [1].

**Infobox Translational Research on Rare Diseases – a funding priority of the Federal Ministry of Education and Research**

A disease is considered rare if fewer than five in 10,000 people are affected by such a diagnosis. More than 8,000 rare diseases are known. It is estimated that more than four million people in Germany alone are affected by a rare disease.

Around 80 % of rare diseases are genetically determined, some diseases cause their first symptoms in childhood. However, the causes of the disease are often unexplored. The relatively small number of people affected, experts and suitable medicines complicate the path to a diagnosis and appropriate therapy. If there is no diagnosis or it can only be made at a late stage, irreversible courses of the disease are often a result. Therefore, research is vital for those affected. Basic research plays an important role here: it not only provides new insights into rare diseases, but can also contribute to a better understanding of more common diseases.

Since 2003, the Federal Ministry of Education and Research (BMBF) has been funding networks that jointly research causes and therapeutic approaches for rare diseases at various university locations. A coordination office supports these networks, among other things, in presenting their results to the public (see also https://www.research4rare.de/wp-content/uploads/2023/05/Poster_R4R_engl_2019-2026.pdf). At the European level, the research consortia are involved in the European Reference Networks (ERN) on rare diseases. In addition, there are international programmes, such as the European Joint Programme on Rare Diseases (EJP RD) for research into the diagnosis and therapy of rare diseases, in which the BMBF also participates.

GAIN (German genetic multi-organ Auto-Immunity Network) is the German network for research and therapy optimisation for individuals with congenital multi-organ autoimmune diseases. The different sub-projects of the GAIN consortium aim to improve the understanding of the development of disease and treatment of individuals with such autoimmune diseases. The GAIN Registry is a central component in the consortium and promotes cooperation between the subprojects, which are described in detail on the GAIN website (www.g-a-i-n.de). These include a biobank coordinated by the Hannover Unified Biobank (HUB) with decentralised sample storage, which supports high-quality biospecimen collection from individuals registered in GAIN. Linking these valuable biospecimens with the associated data from the registry provides optimal support to the research projects in the GAIN consortium.

Patients with immunodeficiencies have been documented for roughly 20 years at the European level in the online registry of the European Society for Immunodeficiencies (ESID). In addition, a national online registry for people with primary (congenital) immunodeficiencies, the PIDNET Registry, was established in Germany starting in 2009. The focus here is on recording individuals with primary immunodeficiencies as comprehensively as possible with the help of a straightforward data set [2]. The GAIN Registry has supplemented the field of existing patient registries for rare immunological diseases since the productive start of online data collection at the beginning of 2021 [3].

## Project

The GAIN Registry builds on the technical platform for documentation provided by ESID and thus guarantees sustainable operation, even after completion of the project period. The GAIN Registry documents genetic, clinical and laboratory data as well as data on therapy and quality of life of individuals with multi-organ autoimmune diseases. Systematic, structured long-term recording by means of regular follow-ups are aimed to gain insights into the incidence and course of the diseases as well as disease-related restrictions on the quality of life.

The GAIN Registry was implemented as a detailed (level 2) data set in the ESID Registry. It is thus available to all ESID centres (https://esid.org/Working-Parties/Registry-Working-Party/Documenting-centers) for documentation. The ESID Registry contains a core data set (level 1), which is also being documented for all GAIN patients. Individuals with a confirmed gene mutation known to cause a multi-organ autoimmune disease can be entered in the registry. However, individuals with multi-organ autoimmune diseases that have not yet been genetically defined can be included as well. All individuals must meet the criteria of the ESID Registry [4]. The decision concerning inclusion in the registry is at the discretion of the attending physician. Patients with an acquired immune deficiency are not included. The most important data source for the registry are the (electronic) patient records. Information originating from local research databases, e.g. on patients’ genetic information, is integrated as well. As a third source, information from questionnaires completed by the afflicted individuals is added to the registry, e.g. on the quality of life issue. No identifying data of the individuals is stored in the GAIN Registry. This data can be merged with the information stored in the registry locally in the documenting centres and according to the rights of use. The documenting centres are clinical institutions such as university hospitals at which the patients are treated. Medical documentalists of the centres transfer the information from the various sources to the registry. For information on data protection, please refer to the section ‘Data protection and ethics’.

## Results and classification

A comprehensive dataset designed and implemented for the GAIN Registry makes it possible to map the complex clinical patterns of people with multi-organ autoimmune diseases. The data structure used is similar to that of an electronic patient record.

Information about afflicted persons that remains unchanged over time is only collected once. Information on laboratory and diagnostic findings as well as questionnaires can be documented repeatedly for each visit to the respective centre. It is aimed to have the included individuals visit and associated documentation in the registry made at least annually. Therapies, infections as well as diseases and other organ-related symptoms are also recorded. For this purpose, it is always possible to specify a start and completion in order to map the course over time. The structure of the GAIN data set can be viewed via a demo version of the ESID Registry (https://cci-esid-reg-demo-app.uniklinik-freiburg.de/EERS, User name: demouser, password: Demo-2019) [5].

To improve its interoperability and sustainability, the GAIN data set is based on international coding systems. This is an important prerequisite to enable comparison or merging with other datasets at a later point in time.

In the GAIN Registry, in addition to the IUIS classification [6] used as a standard in the ESID Registry, the main clinical diagnosis is recorded using the current International Classification of Diseases (ICD-11) of the World Health Organisation [7] and the so-called ORPHA nomenclature. The ORPHA nomenclature is maintained by the European Commission-funded ‘orphanet’ consortium and was developed specifically for rare diseases [8]. The Human Phenotype Ontology (HPO) was chosen for documentation of symptoms [9]. The catalogue is made available directly via an interface (https://hpo.jax.org/api/hpo/docs) in the GAIN Registry to assure that the most current status is always available. Drugs are coded according to their ingredients using the ATC code (Anatomical-Therapeutic-Chemical Classification) [10]. In addition, where possible, the full product name of the drug is documented using a table provided by the European Medicines Agency (EMA). The table contains all medicines approved in Europe [11].

**Figure 1:**
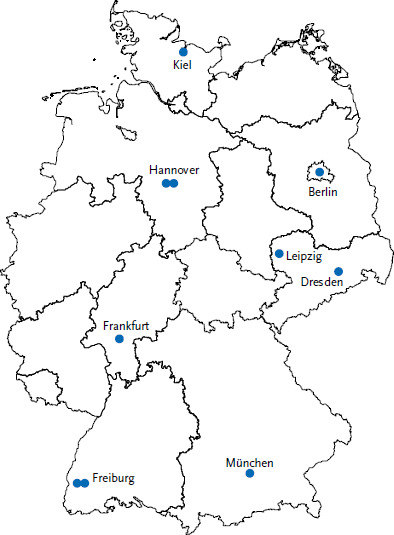
Distribution of centres in Germany documenting in the GAIN Registry. Two documenting centres each are located in Freiburg and Hannover. Source: GAIN Registry

Currently, twelve different centres document in the GAIN Registry, ten centres from various regions within Germany (Figure 1) and one centre each from Milan, Italy and Lisbon, Portugal.

Between 11 January 2021 and 15 May 2023, a total of 486 individuals were included in the GAIN Registry, 258 women (53.1 %) and 228 men (46.9 %). The average age is 39.5 years. If no information on the day or month of birth was available, the middle of the month (15th) or the middle of the year (1st July) was used for the calculation.

A total of 421 afflicted individuals (86.6 %) had a genetic test documented in the registry. At least one gene mutation was detected in 69.6 % (n = 293) of those tested. In total, mutations in 45 different genes were documented. The gene most frequently affected (57 afflicted persons, 13.5 %) is the NFkB1 gene (Table 1) associated with common variable immunodeficiency (CVID). The group of patients with congenital mutations in this gene is the subject of a separate research project within the GAIN network.

Since 2023, the GAIN consortium has been supplemented by an epidemiological study on the quality of life of patients with multi-organ autoimmune diseases and how it relates to patient-oriented care (Qualy-GAIN). Here, the GAIN Registry is an important prerequisite for identifying subjects for the study. Furthermore, in upcoming years, the registry will provide the necessary infrastructure to directly capture not only the questionnaires already used in the GAIN consortium, but also PROMs (Patient Reported Outcome Measures) and PREMs (Patient Reported Experience Measures) related to the Qualy-GAIN study via an app. PROMs collect patient-reported treatment outcomes, e.g. regarding quality of life or health status, by means of standardised questionnaires. PREMS records patients’ experiences during the treatment process, e.g. with regard to care coordination or waiting times. This data will then be linked to the data already present in the registry.

## Key statements

- The GAIN Registry has collected records on people with multi-organ autoimmune diseases since early 2021.
- The aim of the GAIN Registry is to achieve the best possible characterisation of those afflicted in order to gain insights into the incidence and course of the diseases, the course of therapy and disease-related restrictions on quality of life.
- Ten different centres in Germany and one centre each in Italy and Portugal are currently supplying documentation to the GAIN Registry.
- Multi-organ autoimmune diseases can be caused by a mutation in one of many genes; the afflicted individuals in the GAIN Registry currently have mutations in 45 different genes.

## Figures and Tables

**Table 1:** Genes with mutations of those genetically tested in the GAIN Registry (N = 421, n = 217 women, n = 204 men), sorted by incidence.^*^ Source: GAIN Registry

Genes with mutations	Number (N = 421)	%
NFkB1	57	13.5
CTLA4	41	9.7
TNFRSF13B	41	9.7
NFkB2	19	4.5
STAT3	18	4.3
STAT3 GOF (gain-of-function)	18	4.3
LRBA	13	3.1
ADA2	12	2.9
STAT1	10	2.4
PIK3CD	9	2.1
STAT1 GOF (gain-of-function)	8	1.9
IKZF1	7	1.7
ICOS	6	1.4
TNFAIP3	6	1.4
FAS	5	1.2
BACH2	4	1.0
CARMIL2	3	0.7
UNC13D	3	0.7
CARD11	2	0.5
FLG	2	0.5
MST1	2	0.5
PIK3R1	2	0.5
SH2D1A	2	0.5
Other genes	25	5.9
No mutation detected	106	25.2
Results pending	22	5.2

* Only genes documented in more than one individual are listed. There can be multiple mutated genes per each person, which is why the percentages do not add up to 100 %.

## Footnotes

**Data protection and ethics**
The registry is subject to the data protection regulations of the EU Data Protection Regulation (DSGVO) and the Federal Data Protection Act (BDSG) and complies strictly. The ESID Registry complies with the guidelines of the Declaration of Helsinki and has been approved by the Ethics Committee of the University of Freiburg (application no. 493/14 and related amendments) and the local ethics committees of the participating centres. Only patients who provided written consent to participate in the ESID registry can be included in the GAIN registry. They have been informed about the aims and contents as well as about data protection. Participation is voluntary. Consent to participate can be revoked at any time.
